# A novel method to calculate pressure on the twin-tunnel in layered strata

**DOI:** 10.1016/j.mex.2020.101126

**Published:** 2020-11-06

**Authors:** Hai-Min Lyu, Shui-Long Shen, Annan Zhou, Ke-Lin Chen

**Affiliations:** a MOE Key Laboratory of Intelligent Manufacturing Technology, Department of Civil and Environmental Engineering, College of Engineering, Shantou University, Shantou, Guangdong 515063, China; b Civil and Infrastructure Engineering Discipline, School of Engineering, Royal Melbourne Institute of Technology (RMIT), Victoria 3001, Australia; c China Railway Eryuan Engineering Group Co. Ltd., China

**Keywords:** Analytical method, Tunnel pressure, Twin-tunnel, Layered strata

## Abstract

Tunnel pressure from the surrounding rocks plays a critical role for the safety of tunnel. The existing methods for calculate twin-tunnel pressure supposed that the tunnel is buried in a uniform soil layer. This work presents detailed equations of an analytical method to calculate the twin-tunnel pressure in layered strata, which can consider the effects from soil layers. The proposed method is applied to analyse the pressure of the metro twin-tunnels in Chongqing. To demonstrate the efficiency of the proposed analytical method, both the tunnel pressure in layered strata and single strata were calculated. The method article is a companion paper with the original article [1].

• Analyses of the soil parameters;

• Determine the failure pattern A/B;

• Calculate the vertical and horizontal pressure.

## Graphical abstract

**Figure:**
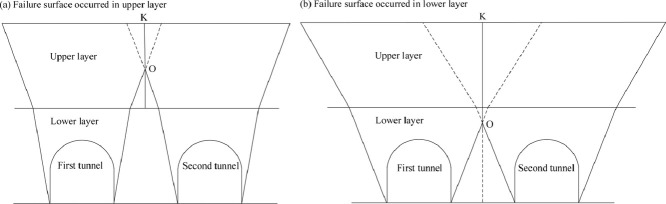

Specifications table

**Table:** 

Subject Area:	Engineering
More specific subject area:	Tunneling and Underground Space Engineering
Method name:	Tunnel pressure calculation method
Name and reference of original method:	Theoretical calculation on rock pressure in shallow-buried twin tunnels [2,3].
Resource availability:	*/*

## Introduction of method

During tunnel construction, it is essential to consider the tunnel pressure from surrounding rocks. The existing methods to calculate the tunnel pressure is always assumed that the tunnel is constructed in a single uniform soil layer [2,3]. However, the tunnel always buried in the complex soil layer. The existing methods are difficult to consider the effects from different soil layers [4], [5], [6]. To overcome the limitation of the existing methods, the proposed method is used to calculate the pressure of a twin-tunnel buried in layered strata. Based on the location of the failure surface, the proposed method is classified into failure patterns A and B. The difference of the failure patterns A and B is the location of the failure surface. The failure surface of pattern A is located in the upper soil layer, while the failure surface of pattern B is located in the lower soil layer. This method article presents a detailed calculation process of tunnel pressure in the layered strata, including method introduction, application, and validation. Tables 1 and 2 list the calculation formulas of tunnel pressures and related parameters for failure pattern A and pattern B, respectively. The detailed derivation process can be found in the original article [1].

**Table 1:** Calculation formula of tunnel pressure and related parameters in failure pattern A.

Parameters of soil layer										
*γ*_1_	*γ*_2_	*θ*_1_	*θ*_2_		*φ*_1_	*φ*_2_	*h*_1_	*h*_2_	*A*	*D*
Judgment criterion of failure pattern A: $d^{\prime }>\frac{2h_{2}}{\tan\varphi_{2}+\sqrt{\frac{\left(tan^{2}\varphi_{2}+1\right)tan\varphi_{2}}{\tan\varphi_{2}-\tan\theta_{2}}}}$										
tan *β*_i_				*λ*_i_						
$\tan\beta_{1}=\tan\varphi_{1}+\sqrt{\frac{\left(tan^{2}\varphi_{1}+1\right)tan\varphi_{1}}{\tan\varphi_{1}-\tan\theta_{1}}}$				$\lambda_{1}=\frac{\tan\beta_{1}-\tan\varphi_{1}}{\tan\beta_{1}\left[1+tan\beta_{1}\left(\tan\varphi_{1}-\tan\theta_{1}\right)+tan\varphi_{1}\tan\theta_{1}\right]}$						
$\tan\beta_{2}=\tan\varphi_{2}+\sqrt{\frac{\left(tan^{2}\varphi_{2}+1\right)tan\varphi_{2}}{\tan\varphi_{2}-\tan\theta_{2}}}$				$\lambda_{2}=\left(1+2\frac{\gamma_{1}}{\gamma_{2}}\frac{h_{1}}{h_{2}}\right)\times \frac{\tan\beta_{2}-\tan\varphi_{2}}{\tan\beta_{2}\left[1+tan\beta_{2}\left(\tan\varphi_{2}-\tan\theta_{2}\right)+tan\varphi_{2}\tan\theta_{2}\right]}$						
$\tan\beta_{2}^{\prime }=\tan\beta_{2}$				$\lambda_{4}=\lambda_{2}$						
$\begin{array}{c} \tan{\beta^{\prime }}_{1}=\sqrt{\frac{1+\tan^{2}\varphi_{1}}{\tan\varphi_{1}-\tan\theta_{1}}\times \left[\frac{1}{\tan(\varphi_{1}-\theta_{1})}+\frac{2h_{1}}{\frac{d}{2}-\frac{h_{2}}{\tan{\beta^{\prime }}_{2}}}\right]}\\ -\frac{1}{\tan(\varphi_{1}-\theta_{1})} \end{array}$				$\begin{array}{c} \lambda_{3}=\left[\frac{2}{h_{1}}-\frac{\left(\frac{d}{2}-\frac{h_{2}}{\tan{\beta^{\prime }}_{2}}\right)\times \tan{\beta^{\prime }}_{1}}{{h_{1}}^{2}}\right]\times \left(\frac{d}{2}-\frac{h_{2}}{\tan{\beta^{\prime }}_{2}}\right)\\ \times \frac{\tan{\beta^{\prime }}_{1}-\tan\varphi_{1}}{1+tan{\beta^{\prime }}_{1}\left(\tan\varphi_{1}-\tan\theta_{1}\right)+tan\varphi_{1}\tan\theta_{1}} \end{array}$						
				$\begin{array}{c} {\lambda^{\prime }}_{1}=\left[\frac{2}{h_{1}}-\frac{\left(\frac{d}{2}-\frac{h_{2}}{\tan\beta_{2}}\right)\tan\beta_{1}}{h_{1}^{2}}\right]\times \left(\frac{d}{2}-\frac{h_{2}}{\tan\beta_{2}}\right)\\ \times \frac{\tan\beta_{1}-\tan\varphi_{1}}{1+tan\beta_{1}\left(\tan\varphi_{1}-\tan\theta_{1}\right)+tan\varphi_{1}\tan\theta_{1}} \end{array}$						
*q*_i_						*e*_i_				
$q_{1}=\left(\gamma_{1}h_{1}+\gamma_{2}H\right)-\frac{\gamma_{1}h_{1}^{2}\lambda_{1}\tan\theta_{1}+\gamma_{2}H^{2}\lambda_{2}\tan\theta_{2}}{a}$						$e_{1}=\lambda_{1}\gamma_{1}h_{1}+\lambda_{2}\gamma_{2}\left(h-h_{1}\right)h_{1}+h_{2}\geq h\geq h_{1}+H$				
$q_{2}=\left(\gamma_{1}h_{1}+\gamma_{2}H\right)-\frac{\gamma_{1}h_{1}^{2}\lambda_{{}_{1}}^{\prime }\tan\theta_{1}+\gamma_{2}H^{2}\lambda_{2}\tan\theta_{2}}{a}$						$e_{2}=\lambda_{{}_{1}}^{\prime }\gamma_{1}h_{1}+\lambda_{2}\gamma_{2}\left(h-h_{1}\right)h_{1}+h_{2}\geq h\geq h_{1}+H$				
$q_{3}=\left(\gamma_{1}h_{1}+\gamma_{2}H\right)-\frac{\gamma_{1}h_{1}^{2}\lambda_{3}\tan\theta_{1}+\gamma_{2}H^{2}\lambda_{2}\tan\theta_{2}}{a}$						$e_{3}=\lambda_{3}\gamma_{1}h_{1}+\lambda_{2}\gamma_{2}\left(h-h_{1}\right)h_{1}+h_{2}\geq h\geq h_{1}+H$				
$q_{4}=\left(\gamma_{1}h_{1}+\gamma_{2}H\right)-\frac{\gamma_{1}h_{1}^{2}\lambda_{1}\tan\theta_{1}+\gamma_{2}H^{2}\lambda_{2}\tan\theta_{2}}{a}$						$e_{4}=\lambda_{1}\gamma_{1}h_{1}+\lambda_{2}\gamma_{2}\left(h-h_{1}\right)h_{1}+h_{2}\geq h\geq h_{1}+H$

**Table 2:** Calculation formula of tunnel pressure and related parameters in failure pattern B.

Parameters of soil layer										
*γ*_1_	*γ*_2_	*θ*_1_	*θ*_2_	*φ*_1_		*φ*_2_	*h*_1_	*h*_2_	*A*	*d*
Judgement criterion of failure pattern B: $d^{\prime }\leq \frac{2h_{2}}{\tan\varphi_{2}+\sqrt{\frac{\left(tan^{2}\varphi_{2}+1\right)tan\varphi_{2}}{\tan\varphi_{2}-\tan\theta_{2}}}}$										
tan *β*_iB_					*λ*_iB_					
$\begin{array}{c} \tan\beta_{1B}=\tan\varphi_{1}+\sqrt{\frac{\left(tan^{2}\varphi_{1}+1\right)tan\varphi_{1}}{\tan\varphi_{1}-\tan\theta_{1}}}\\ =\tan\beta_{1} \end{array}$					$\begin{array}{c} \lambda_{1B}=\frac{\tan\beta_{1}-\tan\varphi_{1}}{\tan\beta_{1}\left[1+tan\beta_{1}\left(\tan\varphi_{1}-\tan\theta_{1}\right)+tan\varphi_{1}\tan\theta_{1}\right]}\\ =\lambda_{1} \end{array}$					
$\begin{array}{c} \tan\beta_{2B}=\tan\varphi_{2}+\sqrt{\frac{\left(tan^{2}\varphi_{2}+1\right)tan\varphi_{2}}{\tan\varphi_{2}-\tan\theta_{2}}}\\ =\tan\beta_{2} \end{array}$					$\begin{array}{c} \lambda_{2B}=\left(1+2\frac{\gamma_{1}}{\gamma_{2}}\frac{h_{1}}{h_{2}}\right)\times \frac{\tan\beta_{2}-\tan\varphi_{2}}{\begin{array}{c} \tan\beta_{2}[1+tan\beta_{2}\left(\tan\varphi_{2}-\tan\theta_{2}\right)\\ +tan\varphi_{2}\tan\theta_{2}] \end{array}}\\ =\lambda_{2} \end{array}$					
$\begin{array}{c} \tan{\beta^{\prime }}_{2B}=\sqrt{\frac{\begin{array}{c} \tan\varphi_{2}\left(1+\tan\varphi_{2}\tan\theta_{2}\right)\gamma_{2}{d^{\prime }}_{2}+\\ 2\left(1+tan^{2}\varphi_{2}\right)\left(\gamma_{1}h_{1}+\gamma_{2}h_{2}\right) \end{array}}{\gamma_{2}{d^{\prime }}_{2}\left(\tan\varphi_{2}-\tan\theta_{2}\right)}}\\ +\frac{\sqrt{\left(tan^{2}\varphi_{2}+1\right)tan\varphi_{2}}}{\left(\tan\varphi_{2}-\tan\theta_{2}\right)}-\frac{1+\tan\varphi_{2}\tan\theta_{2}}{\tan\varphi_{2}-\tan\theta_{2}} \end{array}$					$\begin{array}{c} \lambda_{4B}=\left(2\frac{\gamma_{1}}{\gamma_{2}}\frac{h_{1}{d^{\prime }}_{2}}{h_{{}_{2}}^{2}}+\frac{2{d^{\prime }}_{2}}{h_{2}}-\frac{{{d^{\prime }}_{2}}^{2}tan{\beta^{\prime }}_{2B}}{h_{{}_{2}}^{2}}\right)\\ \times \frac{\tan{\beta^{\prime }}_{2B}-\tan\varphi_{2}}{1+tan{\beta^{\prime }}_{2B}\left(tan\varphi_{2}-\tan\theta_{2}\right)+tan\varphi_{2}\tan\theta_{2}} \end{array}$					
-					$\begin{array}{c} \lambda_{3B}=\left(2\frac{\gamma_{1}}{\gamma_{2}}\frac{h_{1}{d^{\prime }}_{1}}{h_{{}_{2}}^{2}}+\frac{2{d^{\prime }}_{1}}{{h_{2}}^{2}}-\frac{{{d^{\prime }}_{1}}^{2}tan\beta_{2B}}{{h_{2}}^{2}}\right)\\ \times \frac{\tan\beta_{2B}-\tan\varphi_{2}}{1+tan\beta_{2B}\left(tan\varphi_{2}-\tan\theta_{2}\right)+tan\varphi_{2}\tan\theta_{2}} \end{array}$					
*q*_iB_						*e*_iB_				
$q_{1}=\left(\gamma_{1}h_{1}+\gamma_{2}H\right)-\frac{\gamma_{1}h_{1}^{2}\lambda_{1B}\tan\theta_{1}+\gamma_{2}H^{2}\lambda_{2B}\tan\theta_{2}}{a}$						$e_{1}=\lambda_{1B}\gamma_{1}h_{1}+\lambda_{2B}\gamma_{2}\left(h-h_{1}\right)h_{1}+h_{2}\geq h\geq h_{1}+H$				
$q_{2}=\left(\gamma_{1}h_{1}+\gamma_{2}H\right)-\frac{\gamma_{2}H^{2}\lambda_{3B}\tan\theta_{2}}{a}$						$e_{2}=\lambda_{3B}\gamma_{2}hh_{1}+h_{2}\geq h\geq h_{1}+H$				
$q_{3}=\left(\gamma_{1}h_{1}+\gamma_{2}H\right)-\frac{\gamma_{2}H^{2}\lambda_{4B}\tan\theta_{2}}{a}$						$e_{3}=\lambda_{4B}\gamma_{2}hh_{1}+h_{2}\geq h\geq h_{1}+H$				
$q_{4}=q_{1}=\left(\gamma_{1}h_{1}+\gamma_{2}H\right)-\frac{\gamma_{1}h_{1}^{2}\lambda_{1B}\tan\theta_{1}+\gamma_{2}H^{2}\lambda_{2B}\tan\theta_{2}}{a}$						$e_{4}=\lambda_{1B}\gamma_{1}h_{1}+\lambda_{2B}\gamma_{2}\left(h-h_{1}\right)h_{1}+h_{2}\geq h\geq h_{1}+H$				

## Application of the method

The proposed method is used to calculate the pressure on tunnel lining for the Xie-Ao section of Chongqing metro loop line. The detailed information of the twin-tunnel and soil parameters were presented in the main article of this companion paper by Lyu et al (2020) [1]. In the application for real cases, the first step is to determine the failure pattern, which relates to the distance (d) between twin tunnels. The distance (d) can be calculated by the following equation.

$$d^{\prime }=\frac{2h_{2}}{\tan\varphi_{2}+\sqrt{\frac{\left(tan^{2}\varphi_{2}+1\right)tan\varphi_{2}}{\tan\varphi_{2}-\tan\theta_{2}}}}=\frac{2\times 10}{1.0355+\sqrt{\frac{(1.0723+1)\times 1.0355}{1.0355-0.7481}}}=5.31<d=6$$

where *d* is the distance between twin tunnels; $d^{\prime }$ is the affected distance, which is used to determine the failure patter of the twin-tunnel. Since the affected distance is less than the distance between the twin tunnels, the failure surface will occur in the upper layer. Thus, the failure pattern of the twin-tunnel belongs to pattern A. After the determination of the failure patter, the equations list in the Table 1 were applied to calculate the vertical and horizontal pressures. Table 3 lists the calculation results of the Xie-Ao section in Chongqing tunnel. The distribution of the tunnel pressure on the twin-tunnel in the layered strata can be found in the companion research article [1].

**Table 3:** Calculation of tunnel pressure in Xie-Ao section.

Parameters of soil layer									
*γ*_1_ (kN/m^3^)	*γ*_2_ (kN/m^3^)	*φ*_1_ (°)	*φ*_2_ (°)	*θ*_1_ (°)	*θ*_2_ (°)	*h*_1_ (m)	*h*_2_ (m)	*a* (m)	*d* (m)
25.6	24.9	32	46	32 × 0.8	46 × 0.8	7	10	6	6
$d=\frac{2h_{2}}{\tan\varphi_{2}+\sqrt{\frac{\left(tan^{2}\varphi_{2}+1\right)tan\varphi_{2}}{\tan\varphi_{2}-\tan\theta_{2}}}}=\frac{2\times 10}{1.0355+\sqrt{\frac{(1.0723+1)\times 1.0355}{1.0355-0.7481}}}=5.31<6$, (Failure pattern A)									
tan*β*_i_				*λ*_i_					
$\begin{array}{c} \tan\beta_{1}=\tan32^{\circ }+\sqrt{\frac{\left(tan^{2}32^{\circ }+1\right)tan32^{\circ }}{\tan32^{\circ }-\tan{\left(32\times 0.8\right)}^{\circ }}}\\ =3.066 \end{array}$				$\begin{array}{c} \lambda_{1}=\frac{3.066-0.625}{3.066\times [1+3.066\times (0.625-0.479)+0.625\times 0.479]}\\ =0.456 \end{array}$					
$\begin{array}{c} \tan\beta_{2}=\tan46^{\circ }+\sqrt{\frac{\left(tan^{2}46^{\circ }+1\right)tan46^{\circ }}{\tan46^{\circ }-\tan{\left(46\times 0.8\right)}^{\circ }}}\\ =3.768 \end{array}$				$\begin{array}{c} \lambda_{2}=\left(1+2\times \frac{25.6}{24.9}\times \frac{7}{10}\right)\\ \times \frac{3.768-1.036}{3.768\times [1+3.768\times (1.036-0.748)+1.036\times 0.748]}=0.618 \end{array}$					
$\tan\beta_{1}^{\prime }=\tan\beta_{2}=3.768$				$\lambda_{4}=\lambda_{2}=0.618$					
$\begin{array}{c} \tan{\beta^{\prime }}_{1}=\sqrt{\frac{1+0.625^{2}}{0.625-\tan{\left(32\times 0.6\right)}^{\circ }}\times \left[\frac{1}{\tan(32-32\times 0.6)}+\frac{2\times 7}{\frac{6}{2}-\frac{10}{3.768}}\right]}\\ -\frac{1}{\tan(32-32\times 0.6)}=10.611 \end{array}$				$\begin{array}{c} \lambda_{3}=\left(\frac{2}{7}-\frac{0.346\times 10.611}{7^{2}}\right)\times 0.346\times \\ \frac{10.611-0.625}{1+10.611\times (0.625-0.479)+0.625\times 0.479}=0.256 \end{array}$					
-				$\begin{array}{c} {\lambda^{\prime }}_{1}=\left(\frac{2}{7}-\frac{0.346\times 3.066}{7^{2}}\right)\times 0.346\times \\ \frac{3.066-0.625}{1+3.066\times (0.625-0.479)+0.625\times 0.479}=0.126 \end{array}$					
*q*_i_					*e*_i_				
$\begin{array}{c} q_{1}=\left(25.6\times 7+24.9\times 4\right)-\\ \frac{25.6\times 7^{2}\times 0.456\times 0.479+24.9\times 4^{2}\times 0.618\times 0.748}{6}\\ =202.44\text{kPa} \end{array}$					$e_{1}=15.388h-26.00217\geq h\geq 11(m)$				
$\begin{array}{c} q_{2}=\left(25.6\times 7+24.9\times 4\right)-\\ \frac{25.6\times 7^{2}\times 0.126\times 0.479+24.9\times 4^{2}\times 0.618\times 0.748}{6}\\ =235.488\text{kPa} \end{array}$					$e_{2}=15.388h-85.13817\geq h\geq 11(m)$				
$\begin{array}{c} q_{3}=\left(25.6\times 7+24.9\times 4\right)-\\ \frac{25.6\times 7^{2}\times 0.256\times 0.479+24.9\times 4^{2}\times 0.618\times 0.748}{6}\\ =222.469\text{kPa} \end{array}$					$e_{3}=15.388h-61.84217\geq h\geq 11(m)$				
$q_{4}=q_{1}=202.44\text{kPa}$					$e_{4}=e_{1}=15.388h-26.00217\geq h\geq 11(m)$				

## Method validation

To verify the advantage of the proposed approach considering layered strata, the average values of parameters for assumed single stratum were calculated in Table 4.

**Table 4:** Calculation of tunnel pressure in single strata of Xie-Ao section.

Parameters of soil layer						
*γ* (kN/m^3^)	*φ* (°)	*θ* (°)	*h* (m)	*h*_2_ (m)	*a* (m)	*d* (m)
25.25	39	31.2	11	10	6	6
tan*β_i_*			*λ_i_*			
$\begin{array}{c} \tan\beta_{1}=\tan39^{\circ }+\sqrt{\frac{\left(tan^{2}39^{\circ }+1\right)tan39^{\circ }}{\tan39^{\circ }-\tan{\left(31.2\right)}^{\circ }}}\\ =3.372 \end{array}$			$\begin{array}{c} \lambda_{1}=\frac{3.372-1.036}{3.372\times [1+3.372\times (1.036-0.606)+1.036\times 0.606]}\\ =0.225 \end{array}$			
$\begin{array}{c} \tan\beta_{2}=\sqrt{\frac{1+\tan^{2}39^{\circ }}{tan39^{\circ }-\tan{\left(31.2\right)}^{\circ }}\left[\frac{1}{tan{\left(39-31.2\right)}^{\circ }}+\frac{4\times 21}{6}\right]}\\ -\frac{1}{tan{\left(39-31.2\right)}^{\circ }}=5.843 \end{array}$			$\begin{array}{c} \lambda_{2}=\frac{\frac{6}{21}\times \left(1-\frac{6}{4\times 21}\times 3.372\right)\times \left(3.372-\tan39^{\circ }\right)}{1+3.372\times \left(tan39^{\circ }-\tan{\left(31.2\right)}^{\circ }\right)+\tan{\left(31.2\right)}^{\circ }tan39^{\circ }}\\ =0.255 \end{array}$			
-			$\begin{array}{c} \lambda_{3}=\frac{1\times \left(2\times 21-1\times 5.843\right)\times \sin{\left(80.3-39\right)}^{\circ }\times \text{cos}{\left(31.2\right)}^{\circ }}{21^{2}\times \cos\left(31.2+80.3-39\right)}\\ =0.154 \end{array}$			
-			*λ_1_ = λ_4_*			
*q*_i_			*e*_i_			
$\begin{array}{c} q_{1}=25.25\times 11\times \left(1-\frac{0.225\times 11\times \tan{\left(31.2\right)}^{\circ }}{6}\right)\\ =208.363\text{kPa} \end{array}$			$\begin{array}{c} e_{1}=0.225\times 25.25\times 11=62.494\text{kPa}\\ e_{1^{\prime }}=0.225\times 25.25\times 21=119.306\text{kPa} \end{array}$			
$\begin{array}{c} q_{3}=25.25\times 11\times \left(1-\frac{0.255\times 11\times \tan{\left(31.2\right)}^{\circ }}{6}\right)\\ =277.033\text{kPa} \end{array}$			$\begin{array}{c} e_{2}=0.255\times 25.25\times 11=70.826\text{kPa}\\ e_{2^{\prime }}=0.255\times 25.25\times 21=135.213\text{kPa} \end{array}$			
$\begin{array}{c} q_{3}=25.25\times 11\times \left(1-\frac{0.154\times 11\times \tan{\left(31.2\right)}^{\circ }}{6}\right)\\ =230.258\text{kPa} \end{array}$			$\begin{array}{c} e_{3}=0.154\times 25.25\times 11=42.774\text{kPa}\\ e_{3^{\prime }}=0.154\times 25.25\times 21=81.659\text{kPa} \end{array}$			
$q_{4}=q_{1}=208.363\text{kPa}$			$\begin{array}{c} e_{4}=e_{1}\\ e_{4^{\prime }}=e_{1^{\prime }} \end{array}$			

**Fig. 1:**
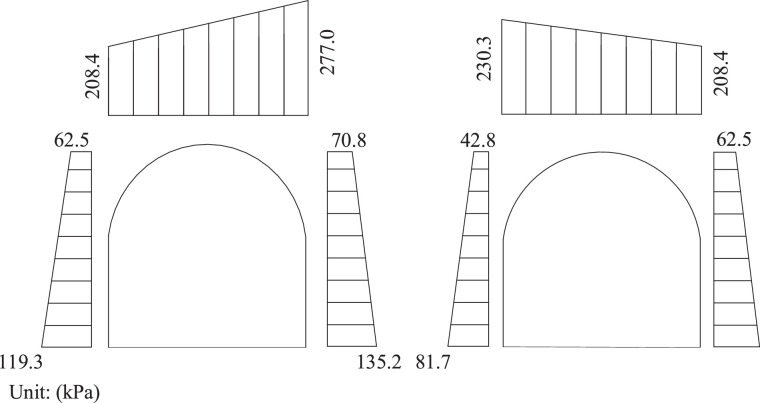
Tunnel pressure of Xie-Ao section in single strata.

Fig. 1 shows the tunnel pressure of Xie-Ao section calculated by assuming a single stratum. As shown in Fig. 1, the vertical pressure is significantly greater than horizontal pressure since the ignorance of different soil layers. The calculated tunnel pressure in the layered stratum can be found in the companion article [1]. The comparison between the tunnel pressure in the single and layered strata indicates that the tunnel pressure calculated by using layered strata is more reasonable than that by assuming a single stratum.

## Declaration of Competing Interest

The authors declare that they have no known competing financial interests or personal relationships that could have appeared to influence the work reported in this paper.

The authors declare the following financial interests/personal relationships which may be considered as potential competing interests:
